# An in vivo assay to study locomotion in Caenorhabditis elegans

**DOI:** 10.1016/j.mex.2022.101890

**Published:** 2022-10-25

**Authors:** Mohamed Abdelhack

**Affiliations:** a Okinawa Institute of Science and Technology, Graduate University, 1919-1 Tancha, Onna-son, Okinawa 904-0495, Japan; b Graduate School of Informatics, Kyoto University, Yoshida Honmachi, Sakyo-ku, Kyoto 606-8501, Japan

**Keywords:** Locomotion, Neuromodulation, Behavioral assay

## Abstract

Adaptation in the sensory-mechanical loop during locomotion is a powerful mechanism that allows organisms to survive in different conditions and environments. Motile animals need to alter motion patterns in different environments. For example, crocodiles and other animals can walk on solid ground but switch to swimming in water beds. The nematode Caenorhabditis elegans also shows adaptability by employing thrashing behaviour in low viscosity media and crawling in high viscosity media. The mechanism that enables this adaptability is an active area of research. It has been attributed previously to neuro-modulation by dopamine and serotonin. This study introduces an experimental assay to physiologically investigate the neuronal mechanisms of modulation of locomotion by dopamine. The technique is utilized to test gait switching while imaging the mechanosensory dopaminergic neurons PDE. Results revealed their role to be not limited to touch sensation, but to sensing surrounding environment resistance as well. The significance of such characterization is improving our understanding of dopamine gait switching which gets impaired in Parkinson's disease.

- *A locomotion pattern switching system was devised to allow studying this process in vivo in the nematode C. elegans.*
- *This system allowed the study of dopaminergic neurons PDE response as the worms switched from crawling to swimming.*

## Graphical Abstract

**Figure:**
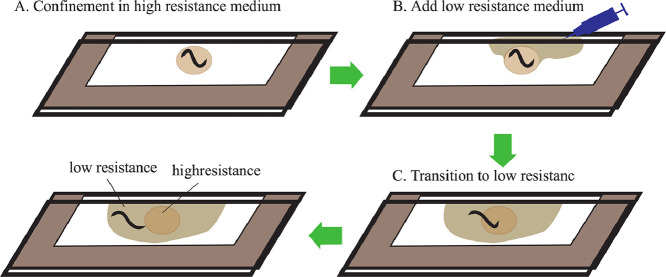

Specifications table

**Table:** 

Subject area	
More specific subject area	*neuromodulation*
Name of your method	*In vivo assay to study gait switching*
Name and reference of original method	*NA*
Resource availability	*Included in the manuscript*

## Method details

### Behavioural assay

**Fig. 1:**
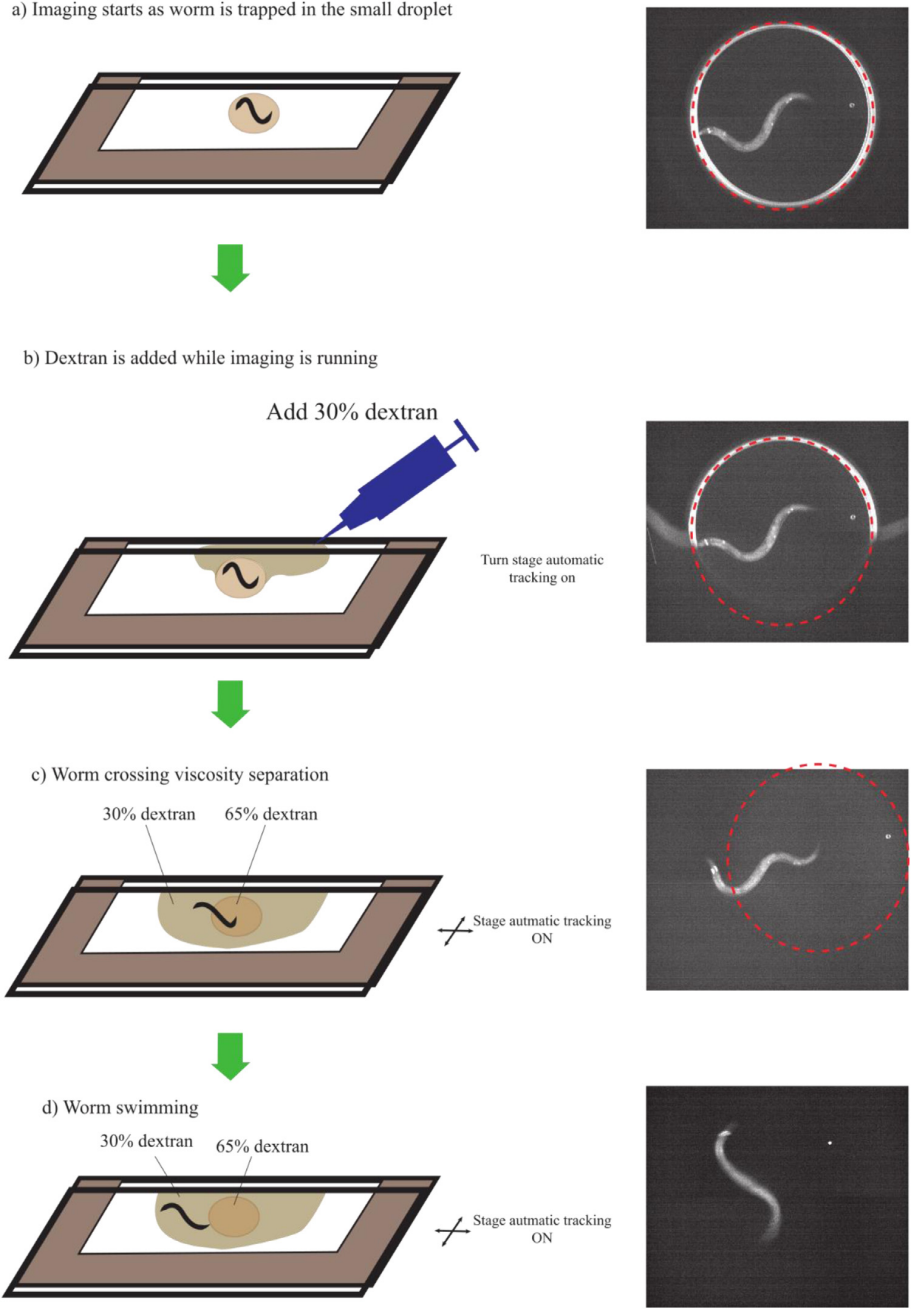
A description of the steps of the viscosity separation experimental protocol where the worm is initially confined in a small droplet of 65% dextran (a) and then dextran 30% is added with a micropipette (zero time point) (b). The worm then starts to move out of the small droplet (c) until10 the whole body gets to the lower viscosity and swimming is maintained (d).

Young adult *dat-1p*::*GCaMP3* worms [4] (Gift from Prof. Dr. David Biron, The University of Chicago) are placed between two glass slides with a paper separator of thickness 0.09-0.15 mm (*n* = 10). The paper is coated with grease to secure the slides together making the resulting thickness become 0.1-0.2 mm (Fig. 1). These dimensions minimize movement along the *z*-axis. The choice of these dimensions will cause the gap between the two plates to be slightly larger than the worm's body diameter. Thus, the wall effects on the normal drag forces will always push the worms to the mid-point between the two plates keeping it always in the focus of the objective lens [6] (except while crossing the gradient where the wall effect would be difficult to calculate). Worms are moved to a foodless plate before assay while the glass cassette is prepared. A tiny droplet (0.2-0.8 µL) of 65% dextran is placed on a glass slide so that the whole droplet is visible under the microscope's field of view. The worm is picked and added to this droplet by a platinum wire worm pick. The second glass slide is then placed on top with the separator in the middle. The top plate is sheared by some distance from the lower plate to ease the addition of the low viscosity medium later. Imaging starts while the worm is confined in the tiny droplet (Fig. 1a). After the imaging starts, 30% dextran is added with a micropipette to fill the area around the 65% dextran droplet, and then tracking starts (Fig. 1b,c). The worm immediately starts moving towards the 30% dextran medium to avoid its confinement and because of the lower resistance. Imaging continues until the whole worm's body passes through the viscosity separation to the lower viscosity region (Fig. 1d). The time taken for the whole worm to fully transfer from the high viscosity medium to the lower viscosity one is variable. However, in all samples presented here, before the last ten seconds shown on the graph, all worms have already passed to the low viscosity media. 30% dextran is used as opposed to pure buffer in order to ease the imaging as the worms swim in this medium but are slower compared to pure buffer. In our test, ten worms were assayed.

The point when the droplet fills in the gaps until half the droplet is chosen to be the zero time point and after that 70 seconds lengths of calcium imaging data are analyzed. Negative controls are obtained through worms that stay confined within the small droplet as the area surrounding is not filled in order to compensate for photo-bleaching (*n* = 10).

#### Signal Preprocessing

**Fig. 2:**
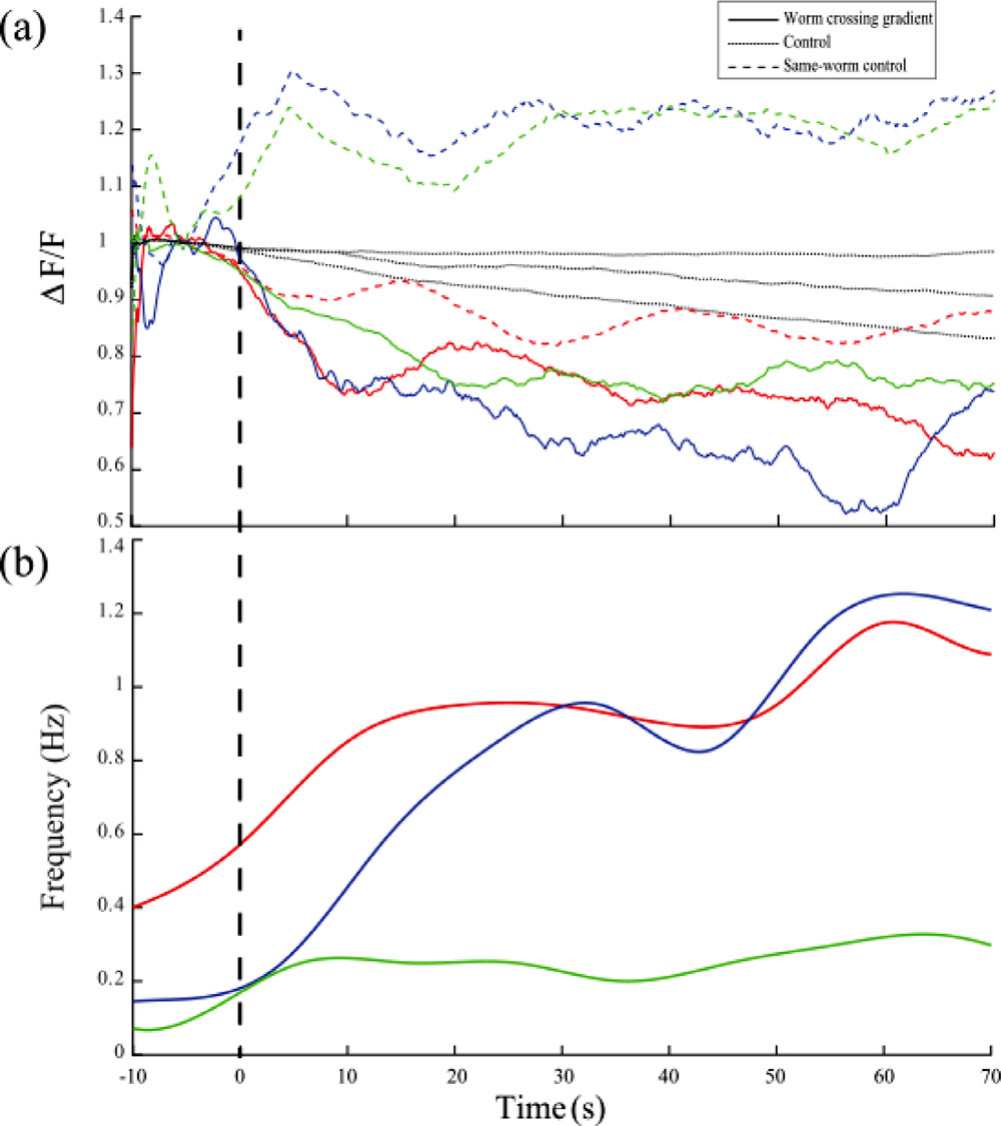
PDE neurons and frequency of undulation response to crossing viscosity separation for three sample worms. Solid lines represent neuronal fluorescence signals of PDE neurons of the worms that cross the separation while dashed lines represent the control signal with mid-body auto-fluorescence signal for the same three worms. Matching colors denote the same worm sample. Dotted lines represent three control worms that remain at high viscosity. Since control worm samples are different so they are not color coded. (b) Undulation frequency responses of the same three worms in (a) as the worms switch from crawling to swimming while crossing the viscosity separation. The frequency was measured every 10 s and then linear interpolation was done to get the smooth curve. The vertical dashed line denotes the zero time point.

Image sequences are imported to ImageJ software [5] and the fluorescent signal is tracked manually using a manual tracking tool [1]. A 30-pixel region of interest around the tracking point is used to extract the fluorescence signal where the mean of the signal in this region is measured. This is a bigger area than the cell size, but it is used to capture blurred signals due to quick movement during swimming. In some cases, the signal from the region of interest is unreliable due to external interference. For example, a deep omega turn by the worm sometimes caused the head dopaminergic neurons, which were also labeled by GCaMP3, to enter the region of interest and interfere with the desired signal. In such cases, the measurement is omitted. Omitted readings are later interpolated using an inpainting function that uses the surrounding time points to create a sparse matrix of spring functions and connections (MATLAB central inpaint nans function). The solution of this function creates a smooth interpolated output (Fig. 2). The signal is then smoothed (Moving-average filter, width=10 s) to remove movement artifacts. Another region of interest not containing the worm is chosen as a background signal that gets subtracted from the neurons’ signal. The average of 10 s before the zero time point is used as a base signal for normalization (Fig. 1).

In order to further ensure the accuracy of the measured calcium signal, another negative control from the same worm was measured. This was obtained by measuring the autofluorescence signal from the worm's body near the midpoint. This area is close to the PDE neurons while not having any neurons labeled by GCaMP3 and would serve as a measure of the effect of movement of the worm in the *z*-axis direction. The mean of the signal from a 50-pixel circular region of interest was measured and normalized using the same method as the neurons’ signals. A bigger region of interest is chosen here because the measured feature is bigger compared to the neurons’ signal and hence the mean of that region is computed, the signal does not require any further normalization.

#### Material preparation protocol

65% dextran was prepared by dissolving 65 g dextran powder 200,000 (Wako Pure Chemical Industries Ltd., Japan) in 100 mL NGM buffer. Mixing was done using the overhead stirrer DLH (VELP Scientifica, Italy). The mixture is then autoclaved to get rid of air bubbles. 30% dextran was prepared by proportional addition of 65% dextran and NGM buffer and then mixing by sonication. For the preparation of NGM buffer, 3 g NaCl + 975 mL H_2_O, 1 mL CaCl_2_, 1 mL MgSO_4_, and 25 mL KH_2_PO_4_ (pH 6.000 adjusted by 5 molar KOH) were mixed after autoclaving to obtain 1 L of the NGM buffer solution. The solution resulting pH was 6.3.

### Imaging

In this experiment, Ca^+2^ imaging was done under Nikon A1R high-speed confocal microscope using its standard widefield fluorescence capability with a 10x objective lens (NA=0.25). The samples were illuminated by a mercury lamp (Nikon Intensilight C-HGFIE) with a GFP filter. The acquisition was done by Andor Zyla 5.5 high resolution camera (Andor Technology Ltd., US) with Micromanager software [2]. Exposure time was set to 100 ms so the resulting frame rate is 10 FPS and 4 × 4 binning was done on each image so that the final resolution is 640 × 540 pixels. The resulting axial resolution is 8.5 µm which is above the typical size of a *c. elegans* neuron cell body thus capturing the signal from the whole cell body with a small tolerance for small z-axis movements. Furthermore, the choice of region of interest size described in the previous section further increases the effective slice thickness making measurements even more resistant to *z*-axis movements. Autotracking was done using Hawkvision movable stage system (Hawkvision Co. Ltd., Japan) equipped with Point Grey GRAS-03K2M-C bright field camera (Point Grey Research Inc, Canada). Tracking software is controlling the stage movements in order to keep the whole body of the worm in the field of view [3].

#### Data analysis

As the worms cross the viscosity separation (Fig. 1), PDE neurons have shown decreased activity responses (Fig. 2). The decrease is shown not to be a result of either photo-bleaching or loss of focus due to *z*-axis movement as both controls showed a very small decrease in activation compared to the neuronal signal of the worm crossing the viscosity separation. The frequency of head undulation of *C. elegans* increased accordingly, however even if it does not increase to reach the swimming typical frequency (Fig. 2b), the corresponding PDE response was reduced nonetheless. This suggests that PDE neurons respond by a decrease in activation to the environmental pressure and not to the body bends. However, they still showed a response to body bends which was smoothed out.

**Fig. 3:**
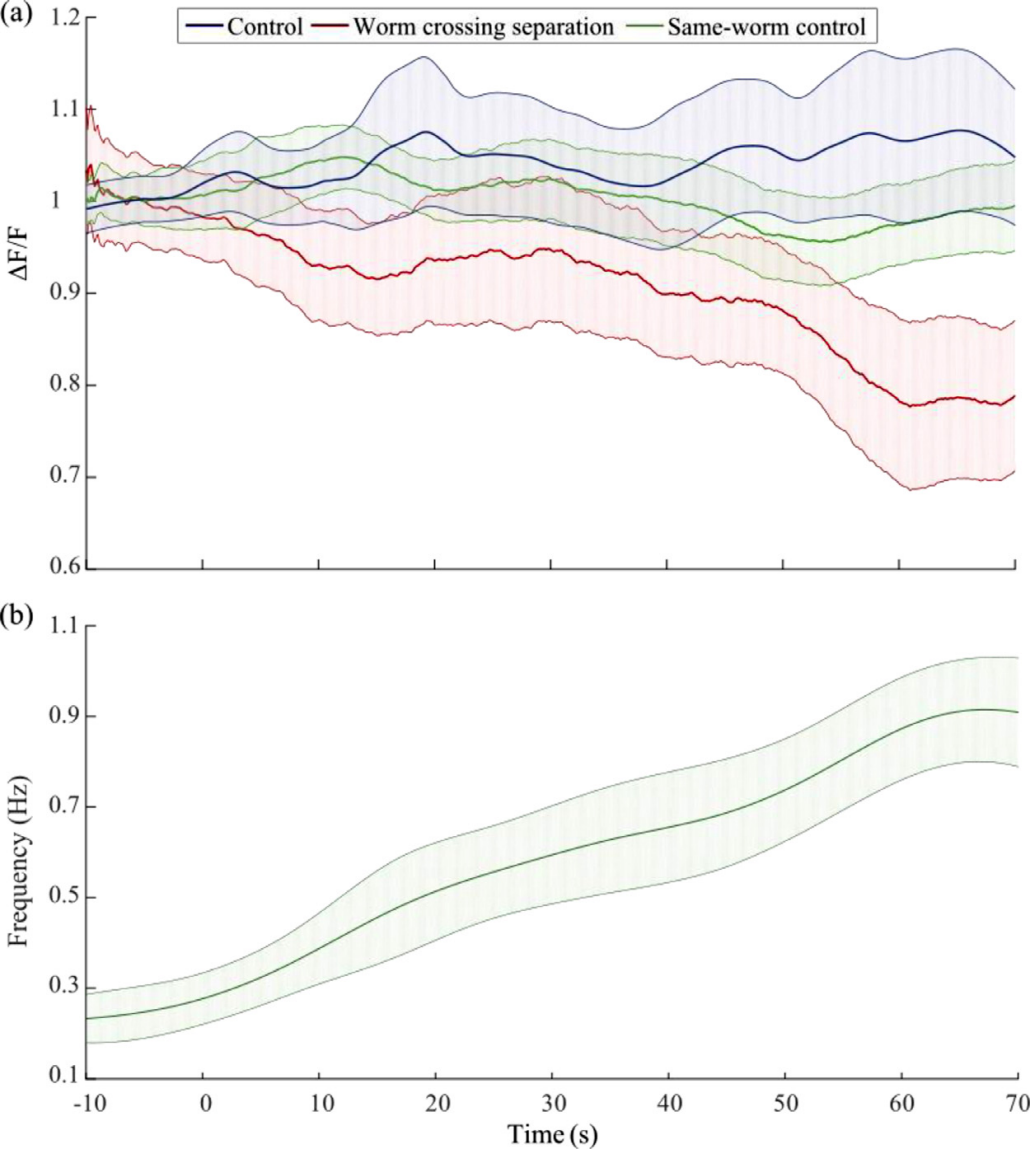
Mean fluorescence signal of PDE neurons and corresponding behavioural response to the worm crossing viscosity separation: (a) Average PDE response to crossing the separation where the zero point is the point of addition of the droplet of liquid (*n* = 10). The control represents worms that remain at high viscosity (*n* = 10). Same-worm control is the measurement of auto-fluorescence signal from the mid-body in order to ensure that the measured decrease in activation is not due to loss of focus. (b) Average frequency response to crossing the separation of the worms in (a). Error bars correspond to ±2 SE.

The mean calcium response to crossing the separation is shown in Fig. 3 where zero time point refers to the time when 30% dextran is visible to have surrounded half of the 65% dextran droplet (Fig. 1b). I show also the response of up to 70 s after the zero time point since the worm's body, and hence the PDE neurons, takes 10-60 s to fully move from the 65% dextran and be fully swimming in 30% dextran which is apparent in the gradual increase in undulation frequency (Fig. 3b). The transition time is variable among worms, so the mean calcium signal shows gradual change until it stabilizes at about 60 s to about 75% of its value in high viscosity. In some cases, we observe a sudden drop while in other cases the drop is gradual (Fig. 2). This depends on the speed the worm crosses the separation which is variable. When the worm takes more time to pass the gradient, the sensillar endings of PDE neurons move through the viscosity gradient more slowly and hence the calcium response goes down more gradually.

**Fig. 4:**
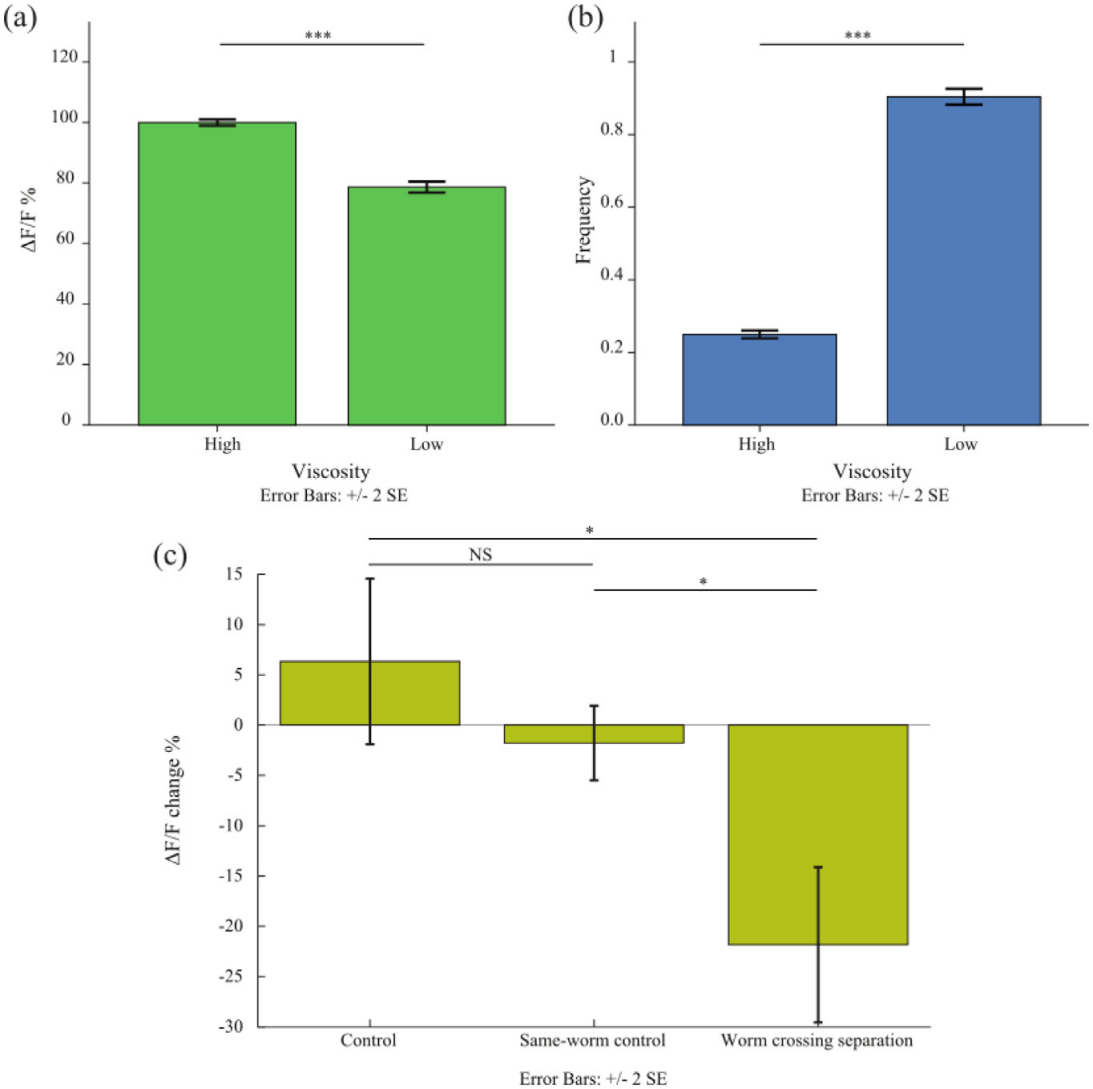
Comparison of mean of data from 10 s before the zero time point and from 60 to 70 s after zero time point where the worm in each case is in a completely homogeneous viscosity. (a) Mean activation of PDE neurons in high and low viscosities shows a significant difference. (b) The mean frequency of undulation also shows two different frequencies that are characteristic of both crawling in the high viscosity case and swimming in the low viscosity case. (c) The mean difference in fluorescence signal of the PDE neurons in the worm crossing separation in comparison to the mid-body auto-fluorescence signal and to the PDE neurons signal from the control worms that remain in the high viscosity medium. It shows that the difference in the observed decrease in activation is a result of the physiological change associated with sensing the environment and not photobleaching or loss of focus.

The separation described is also meant to be a very steep gradient of viscosity but as time passes and due to diffusion and stirring effect caused by the worm movement, the gradient can get smoother, and hence the transient in the physical forces sensed gets smoother. This can explain why the fluorescence signal drop is more gradual than expected in some cases. For this reason, in Fig. 3a, the mean signal of the PDE neurons is close in level to the controls until the last 10 s when all the worms have moved completely to the low viscosity medium. This could also be attributed to the slow response of these neurons. The ADE neurons, which are similar in genetic identity to the PDE neurons, have been shown to exhibit a slow response to harsh touch stimulation [4]. The signals from 60 to 70 s were compared to signals from 10 s before the zero time point and are significantly different (Fig. 4) (*p* < 0.001, Unpaired two-sample t-test) where the frequency of undulation, as expected, is also significantly different (*p* < 0.001, Unpaired two-sample *t*-test).

The transition period could have unexpected signal changes due to many reasons including z-axis movement and stirring effects. Thus, it was important to compare the signal level between worms undulating in homogeneous media. Hence, the mean difference between the PDE signals from 60 to 70 s and signals from 10 s before the zero time point was compared for the PDE neurons crossing the separation and across the two controls (Fig. 4c) The PDE neurons’ signals are shown to decrease by 21.4% on average after completely crossing the viscosity gradient (60 to 70 s after zero time point) compared to its signal in high viscosity medium (10 s before the zero time point). This decrease is significant compared to a decrease of 1.7% in the same-worm auto-fluorescence signal (*p* = 0.0211, Mann-Whitney *U* test) and to an increase of 6.3% in the worm in high viscosity neuronal signal (*p* = 0.0211, Mann-Whitney U test). On the other hand, the signal change compared between the controls did not show any statistically significant difference from each other (*p* = 0.7337, Mann-Whitney *U* test) indicating either minimal *z*-axis movement or no effect on signals collected using the widefield fluorescence microscopy due to z-axis movements. This indicates the success of the used procedure in measuring the physiological change in the PDE neurons due to crossing the viscosity separation. In this protocol, 10 worms were utilized which was sufficient statistically given the strength of the effect but it could be possible to increase the sample count to decrease the effect of variance in the speed of crossing the separation.

## Ethics statements

*Experiments were conducted under the ethical guidelines of the Okinawa Institute of Science and Technology.*

## CRediT authorship contribution statement

**Mohamed Abdelhack:** Conceptualization, Methodology, Formal analysis, Data curation, Writing – review & editing.

## Declaration of Competing Interest

The authors declare that they have no known competing financial interests or personal relationships that could have appeared to influence the work reported in this paper.
